# From Our Correspondents

**Published:** 1985-01

**Authors:** 


### From Our Correspondents

### Orthopaedic Developments in Bristol

Important decisions have been taken which must enhance progress in orthopaedics and improve service to patients in Bristol.

Professor Louis Solomons has been appointed to the Chair of Orthopaedics in Bristol. It was in 1980 that an appeal was launched to establish this chair. The vigour of its protagonists and the active support of its Patron, H.R.H. the Prince of Wales, resulted in the collection of the target sum of half a million pounds in two years. In 1982, Mr. David Fuller was appointed as the first Professor of Orthopaedics to start in September 1983. Sadly this was not to be. On 16th July 1983 the helicopter on which he was travelling with his entire family crashed on a flight to the Isles of Scilly and they were all killed.

Professor Solomons already holds the Chair of Orthopaedics at the University of Witwatersrand in Johannesberg. He is a South African, was the first professor and built up the department there. He is co-author with Alan Apley of St. Thomas Hospital, London of a standard textbook of orthopaedics and will come to Bristol with a international reputation and the authority of experience in an academic chair.

The bulk of 'cold' orthopaedic surgery in Bristol is carried out at Winford Hospital, opened with 56 beds in 1930 as an open air hospital and school for crippled children. In 1939 a new block of 160 beds was built under the Emergency Medical Service for the admission of adult orthopaedic patients. This hospital, of necessity designed and built under wartime conditions has already long outlasted the expectations of its planners and its replacement is long overdue. The rural location originally designed for the open air treatment of tuberculous patients now only results in inconvenience and expensive travelling for visitors and staff. After consideration of all the alternatives the Southmead Hospital site has been chosen. The new Orthopaedic Department will be a 3 storey building with 140 beds and three operating theatres. It is estimated that it will cost £11 million and take 7–8 years to complete. The new Professor will no doubt have much influence on the detailed planning. He will need patience as well as drive to sustain him. The final outcome could be one of the finest orthopaedic centres in the country.

H.E.D.G.
W.J.A.
M.G.W.

### Mastership Restored

The University of Bristol has just restored its Mastership of Surgery. The ChM degree was withdrawn about a dozen years ago at the instigation of a previous Professor of Surgery. One or two other universities (Glasgow, Birmingham) had abolished their masterships at about the same time, believing perhaps that one higher degree (an MD) was adequate for a medical faculty (the PhD is a different kettle of fish, being a supervised degree and one that is by no means restricted to medical graduates).

Locally the loss of the ChM proved unpopular. When the matter was raised at a meeting of the University Surgical Teachers two years ago, there was near-total support for the suggestion that it be resuscitated. Following a petition to the Board of Medical Studies the proposal was adopted by the Senate and Council of the University, and the degree has been re-introduced to the statute books.

The new degree is a little different to its predecessor. As before it is accessible both to graduates of the University of Bristol and to those of most other universities who carry out their research in Bristol, subject of course to the title and outline of the thesis being accepted and to a satisfactory examination of the completed work. However, since the term "Master of Surgery" conveys the expectation of some clinical competence in the subject, it will usually be necessary for candidates whose thesis is accepted to pass a short clinical examination as well. This examination may be taken either in general surgery or the relevant specialty (e.g. otolaryngology, orthopaedics); it will be waived in the case of those who are accredited for higher surgical training. A clinical examination is also a feature of some other surgical masterships, notably the Cambridge MChir in which it is unavoidable. In Bristol it will to some extent be counterbalanced by the fact that external candidates are required to work in this University for a minimum or one year as opposed to two years for the MD. Nonetheless, that regulation should not be construed as indicating that the ChM thesis is inferior to an MD. The process of appointing assessors, an adviser and examiners (two external and one internal) will be identical, and a viva voce examination will be customary.

At present few if any trainees in general surgery can obtain a senior registrar post, let alone a consultant post, without having undertaken a period of formal scientific research. Although the desirability of this requirement is arguable, the fact itself can scarcely be disputed. Moreover, the practice may well extend to the major surgical specialties. In this context it matters little whether the trainee surgeon is working towards a mastership of surgery or a doctorate of medicine. Surgeons generally obtain the one (where available) and physicians the other, though there are subtle differences. A candidate sitting for a

ChM can generally expect to find that at least one of his thesis examiners is a surgeon, but this is not necessarily the case if he takes an MD (especially in basic sciences). By contrast a mastership may not carry quite the same cachet as a doctorate, and the gown and cap are of muted splendour. The situation is anomalous, since many doctors would regard a mastership of surgery as being senior to a doctorate of philosophy. At least one Australian university offers a doctorate of surgery and perhaps that is an example that Bristol should follow. Meantime the choice of higher degree is once again available to Bristol surgeons, and academic masochists can strive to bag a brace.

R.C.N.W.

**Management Budgeting Trial**

Southmead Hospital has been involved in this trial together with several other hospitals in the country for over a year now. Essentially, the aim is to cost the expenditure of seven clinicians in the hospital for a year. The hope is that this should give a more accurate prediction of expenditure by the district in future years, and allow for variance for new techniques of treatment. After preliminary discussions and costings of items, such as blood tests, theatre time, use of physiotherapy, drugs etc., the pilot study started on 1st April. So far, each of the five surgeons and two physicians involved has received analysis for five months.

The guesstimates which we made of duration of stay, use of diagnostic and therapeutic services, have proved to be reasonably near the mark. Not surprisingly, diagnoses in medical wards have proved to be wide ranging, and the thirteen commonest diseases only comprise three fifths of the conditions treated. Of interest has been the high number of blood investigations done out of hours (each of which is charged £5 extra), but this is a reflection of the large number of emergencies treated by physicians, most of which are admitted outside the nine-till-five Monday-to-Friday hours which our administrators work. The frustrating aspect of the figures is the high proportion (six sevenths) of fixed costs – salaries, feeding, heating, etc., over which the clinician has no control. I have calculated that I can influence only £120 per patient stay and £6 for each out-patient visit. These sums have to cover all diagnostic and treatment services provided by my firm.

It may be that after a year, we shall be able to see trends of expenditure, and compare costs of clinicians in the same hospitals or in different parts of the country. There may be an economical dividend in procedures under the control of our nurses and junior staff; for example, one ward sister has labelled all disposables in the ward with their costs. But we have no control of the type of patient admitted – a recent patient with Legionaire's disease cost £20,000 as he needed ventilation in the I.C.U. for several weeks. It is clearly desirable that all health service staff become cost conscious, but quality of care is difficult to measure and seldom figures in management statistics. What appals me about clinical management budgeting is the cost of the exercise – £250,000. If the pilot study is fully evaluated before wide application, it might prove worthwhile, but present indications are that the D.H.S.S. is wanting all hospitals to work on this sytem before we know whether it really is worthwhile. With the recent reorganisation only just being digested, the imposition of managers and now of management budgeting seems to me to risk severe administrative dyspepsia.

H.G.M.

**S.O.S. – Twenty First Birthday**

Born in 1963, the Southmead Orchestral Society is now 21 years old. In its early stages it met in a private house. Since 1968 it has met on the third Saturday of every month at Southmead Hospital. At present it has 66 members and usually musters about 45 players at a session. An attendance record had been kept since January 1965 and of the 19 players then present six are still regular members. The orchestra has a strong link with this journal because three members have been its editor – Dr. Norman Brown, Dr. David Reeves and the present editor who has also been secretary of the Society since its inception. Of course there have not been enough instrumentalists on the staff of Southmead Hospital to make up a symphony orchestra, but friends and friends of friends have joined in. Nevertheless 47 doctors have played with us, including 16 consultants, 14 junior hospital doctors and 17 general practitioners, also eight nurses and physiotherapists, 4 technicians and one administrator. It all began when Dr. Irene Gibbs, a paediatrician, invited a little party to her house to play Brandenberg Concertos. Her husband David, a Senior Lecturer in Physics at Bristol University (whose speciality is semi-conductors) is a versatile musician and took on the job of conducting us. His skill, coupled with tact and humour, has been one of the main ingredients in keeping us going. Our object has been to enjoy playing music together and not to perform publicly, and to make each session a sort of party with half an hour's break for food and drink and socialising. Another main ingredient in our survival is our venue, – Monks Park Hall and we most gratefully thank the administration of Southmead Hospital for allowing us to use it and the Catering Staff for providing us with food.

Our nearest approach to a concert is our annual event in June, when we go to the Bishop's Palace at Wells and play in idyllic surroundings. Players invite an audience of friends and relations who are under no illusions about the quality of the performance they are about to hear, and who are compensated for their forbearance by being given dinner afterwards at the Swan Hotel. The most memorable of these occasions for us was when Rosemary Bickersteth, the wife of the Bishop of Bath and Wells and at one time a concert pianist, did us the honour of playing Beethoven's fourth piano concerto with us. Never have we rehearsed so assiduously. She played it marvellously and carried us all along to unexpected heights. On another memorable Wells accasion we re-enacted the first performance of Haydn's Farewell Symphony. Haydn had wished to remind his master, Prince Esterhazy, that the orchestra were long overdue for their annual leave. In the last movement he directed individual players at intervals to snuff out their candles and tiptoe off, until, at the very end, only the conductor – Haydn himself, and one other player were left on the stage. It is recorded that the Prince *was* amused and that they got their leave. We dressed ourselves in eighteenth century costumes from the Old Vic wardrobes and equipped ourselves with candles. Whatever the verdict on our playing everybody agreed that the ladies looked quite breathtaking.

During our 21 years we have given ourselves the experience of playing most of the great classical symphonies, some of several times – e.g., Beethoven 1—8, all four of Brahms, most of Schubert, Tchaikovsky, and Dvorak, all the later symphonies of Haydn and Mozart that are scored for full wind, we have even had a go at Mahler, Bruckner, Sibelius and Rachmaninoff. If our members want to play concertos they have the opportunity, guest artists have come to play with us and we have got through (sometimes scraped through) the great concertos of Beethoven, Brahms, Rachmaninoff, Schumann, Grieg, Saint Saens etc and enjoyed every moment of it. We follow Chesterton who said 'If a thing's worth doing, it's worth doing badly!?

Wagner wrote his Siegfried Idyll to celebrate the birth of his son. Our twenty-first birthday has been celebrated with the composition of a Southmead Idyll by Nigel Dodd. It is a lovely work composed with our limitations in mind and with a bit of 'jam' for everybody, It is however, worthy of a better performance than we can give it. One day we hope to hear a 'real' orchestra play it. It could catch on and enter the repertoire, then indeed our existence will not have been in vain.

M.G.W.

**Whither Taxation: Further Education?**

When young it was easy to wonder why father moaned about his tax. Now it is not only a question of wonderment. More perplexing, maybe, and certainly less understandable.

Recently many parents of potential university students will have noted with unseemly interest Keith Joseph's proposed rises in parental contribution to Students' living costs, travel and even to the educational fees themselves. Such changes are clearly signs of the times: self-sufficiency, privatisation and all that. Well and good you may say, but at least they are logical.

Or are they? Happily even medical parents do not often spawn a genetically homogenous clone of university students. Most broods are a mixture. Some are academically bright; some want to fend for themselves. However jobs are not necessarily easy to obtain these days for the recent school leaver. Frustrating for these school leavers though it may be, there is in the background Social Security and its Safety Nets. Amazingly in this field there are no parental means tests. The young just need to leave home and financial support is not their parent's worry any more. Dole (admittedly not generous, but at least, better than domestic pocket money), clothing allowances and rental for a flat follow the Official Forms and short-term patience. Well-informed sources tell me that this support now amounts to at least £3000 per annum per offspring.

Robbing Peter to pay Paul? Perhaps. I just get the feeling that some of us could be paying both Peters and Pauls. Is that what privatisation really means? Are we paying taxes for our own unexpected Further Education?

